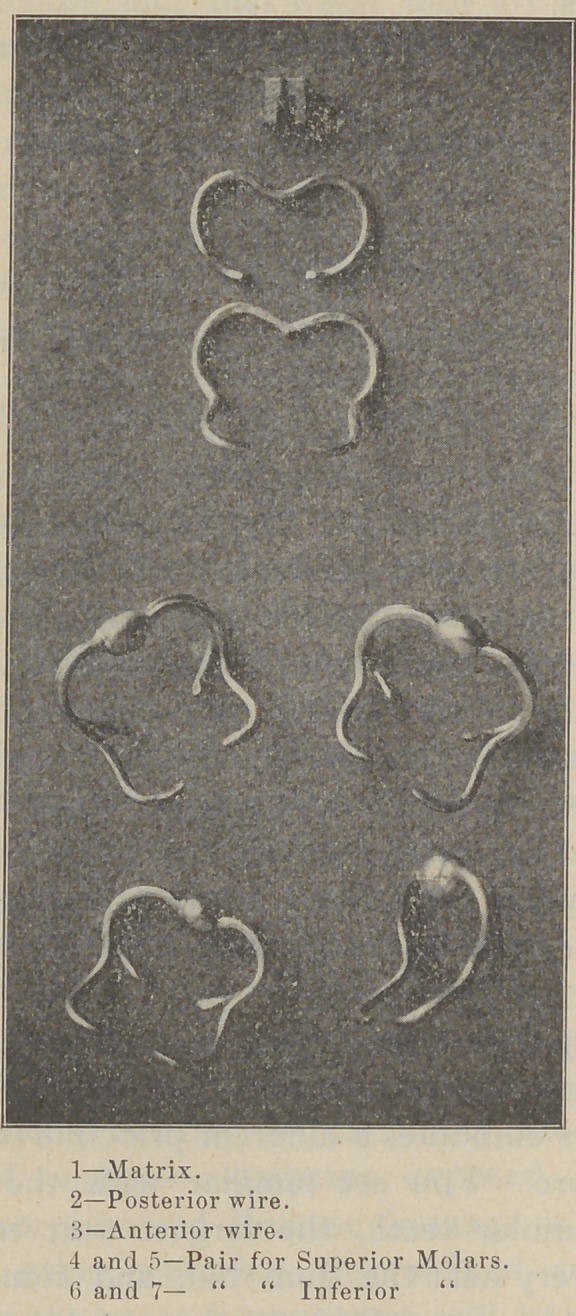# A New Form of Clamp

**Published:** 1900-03-15

**Authors:** H. F. Harvey

**Affiliations:** Cleveland, Ohio


### A NEW FORM OF CLAMP.

BY H. F. HARVEY, D.D.S., CLEVELAND, OHIO.

A Talk before the Ohio State Dental Society, December, 1899.

I have no paper to read and very little to say, so that I will not detain you long. The program calls for a new form of clamp. Sometimes we present something which we think is very new and we find it to be quite old.

We have many clamps on the market very well adapted to the ordinary forms of teeth, but occasionally they are utterly useless. I think all of us have had the experience of finding teeth on which it was very difficult to get any clamp to hold and retain the rubber satisfactorily, especially those molar teeth which are not thoroughly erupted or are very conical in shape and have many abnormal forms.

The clamp I have been using in such cases is one of my own design and has given some satisfaction, and I think perhaps embodies a different principle than has been applied before. You are familiar with the anatomical form of the molar teeth, the largest part taken bucco-lingually is very near the gum line and sometimes it is even below this, especially in teeth not fully erupted. In these cases it is necessary to force the ordinary clamp down quite a ways to get it to hold and sometimes then we are not able to do it, and all of this is not very pleasant to the patient.

If you examine the tooth from the other direction, mesio-distally, you will find the crown quite bell shaped,

1—Matrix.
2—Posterior wire.
3—Anterior wire.
4 and 5—Pair for Superior Molars.
6 and 7— " " Inferior "

the largest diameter being near the occlusal surface, and if you can get a clamp which reaches around the angles of the tooth and get a grip mesio-distally it will hold without going so low, and that is the principle utilized in this form of clamp.

This clamp instead of being made of one piece of flat steel, as they usually are, is made of two wires of different sizes and united in the center of the bow, as I will show you.

I have used different materials, German silver, steel wire, etc., united together with soft and hard solder but lately I have adopted piano wire, soft solder to bind together, thus retaining the temper of the wire which is better than any I am able to give it.

These drawings will give you an idea of the clamps and their separate parts. The anterior wire is heavier than the back one, because the leverage is greater, and you need a stronger spring. The points are bent around to grasp the tooth in the mesial and distal angles. These points adjust themselves until the forces are equalized where the force or pressure of each point will be directly toward the center or long axis of the tooth. Each point acts independently of the other, so you do not get any rocking of the clamp, the grip being at the extreme ends. Also if you force the clamps down they are not so painful as other clamps where you have a single surface. This form of clamp is not as well adapted to teeth having large approximal cavities, unless the points can be carried below the cavity.

The size of anterior wire is .043 in. or 19 English gauge.
" " posterior " .036 " 20 " "

In bending the wires to proper form I use a pair of ordinary bending pliers, first making the depression in the middle of bow, and continue the bending each way, finishing at the points.

It is necessary to slightly flatten both surfaces of wires where they come to a point for this purpose also, as it gives greater bearing surface. The points that come in the matrix should be of steel, and I make them by filing out of an old excavator handle.

The wires must fit closely (tight) in the matrix depressing on the soft solder only to hold parts in position, and being careful that it does not flow far from matrix. The matrix is finished up after soldering. The tension of clamp may be varied somewhat by the size of the bow as well as size of wires.

I have here a number of teeth set in plaster which vary considerably in form and size, also one of the clamps. I will pass them around with the clamp forceps so you may see the clamp and its adaptability to various forms of the teeth.

DISCUSSION.

Dr. C. R. Butler, Cleveland, Ohio: I have a few words to say in commendation of the clamps which Dr. Harvey, in his modesty, has presented. There is a principle embodied that I have not found in any other form of clamps. I have put them to a practical test, and find that there is adaptation of considerable range. These presented are for the molar teeth, but they may be made to use for bicuspids as well.

Dr. H. L. Ambler, Cleveland, Ohio: The Harvey clamps are both unique and practical. They are unique because they are made from piano wire, and as we often say of many inventions when we see them, "It is a wonder that some one did not think of them before," but it remained for Dr. Harvey to produce this invention, notwithstanding dentists and manufacturers have been making clamps for thirty years. They are practical because they have a bearing on the teeth, both mesio-distally and bucco-lingually, owing to their peculiar construction, and also because with a pair of pliers they can be slightly changed in form and made to fit a little larger or a little smaller tooth, or an abnormally shaped one. In cases of partially erupted molars, they will hold on better than any clamp with which we are familiar.

Dr. Henry Barnes, Cleveland, Ohio: Dr. Harvey loaned me his clamps for a few days, also explained how they were made. They are simple of construction, yet difficult to shape at first. They are the best form of clamp yet devised in that they are automatic in their action, out of one's way, especially when operating upon cervical cavities in the molars. They can be used when no other clamp will hold, and are not as painful as are other forms.

Dr. W. T. Jackman, Cleveland, Ohio: Through the kindness of Dr. Harvey I was permitted to try this new clamp on a conical, superior right second molar, a tooth on which I used or tried to use an exceedingly convex (gumward) clamp and one almost stiff enough, apparently, to crush the tooth but it would not hold. With the Harvey clamp I adjusted the rubber, used a dam holder with three points of attachment, drawing the corner of the mouth well back, yet the clamp held nicely till I inserted three crown cavities, and, to my surprise on removing it, it seemed to have but slight hold; but it clamped in such a way that slipping was impossible. It will no doubt prove to be an excellent aid to the armament of the operator.

Dr. J. F. Stephan, Cleveland, Ohio: Having had the opportunity during the past few weeks of using the clamps Dr. Harvey has shown us, I would like to say a word in appreciation of their good qualities. They certainly fill a long-felt want in being so nearly universal, and especially in fitting those cases where the crown is only partially erupted. In these cases the mesio-distal force is brought into play, and it is surprising how firm they hold exerting as little force as they do. I think it will be impossible to injure the enamel of a tooth using one of these clamps. The fact they can be bent so as to fit the crown of malformed teeth is a valuable feature and one which will certainly be appreciated. I believe they deserve special notice because they will help us when all other clamps fail.

Dr. Fletcher, Cincinnati, Ohio: For one, I feel very grateful to Dr. Harvey for having presented to us what seems to meet a long-felt want in the principle of making clamps, for what he has produced certainly must be efficient and satisfactory, and I wish to thank the Doctor for what he has done in that line.

## Figures and Tables

**Figure f1:**